# Comprehensive Single-Cell RNA Sequencing Analysis of Cervical Cancer: Insights Into Tumor Microenvironment and Gene Expression Dynamics

**DOI:** 10.1155/ijog/5027347

**Published:** 2025-05-13

**Authors:** Xiaoting Shen, Huier Sun, Shanshan Zhang

**Affiliations:** Department of Gynaecology and Obstetrics, The First People's Hospital of Linping District, Hangzhou City, Zhejiang Province, China

**Keywords:** cervical cancer, differential expression, gene coexpression network, pseudotime analysis, single-cell RNA sequencing

## Abstract

**Background:** Cervical cancer is a complex disease with considerable cellular heterogeneity, which hampers our understanding of its progression and the development of effective treatments. Single-cell RNA sequencing (scRNA-seq)—a technology that enables gene expression analysis at the cellular level—has emerged as an important tool to explore this heterogeneity on a cell-to-cell basis. We perform an analysis on data quality and differential gene expression in cervical cancer via scRNA-seq, giving insights into the tumor microenvironment and likely therapeutic targets.

**Methods:** scRNA-seq for cervical cancer sample and advanced bioinformatics tool for data analysis were utilized. Scatter plots were generated to assess quality control metrics based on mitochondrial gene expression and total RNA count. Cell clustering differential expression analysis identified significant genes in each cell cluster. Gene coexpression networks and modules were performed network analysis. We utilized pseudotime analysis to model the experience of cell state transitions to infer a trajectory and functional enrichment analysis to understand the biological processes involved.

**Results:** scRNA-seq data revealed distinct cluster pattern of high quality gene expression profile. Ultimately, differential expression analysis suggested significant genes: TP53, GNG4, and CCL5 had high degrees of differential expression and potential roles in tumor progression. Some of these gene modules have unique biological functions identified by network analysis, while dynamic changes in gene expression across the trajectory of the pseudotime reveal the differences in gene expression during cell state transition. We next performed functional enrichment analysis which revealed that immune response and metabolic processes play a pivotal role in cervical cancer.

**Conclusion:** Our large scale scRNA-seq of cervical cancer provide insights into cellular heterogeneity and gene expression dynamics within the tumor microenvironment.

## 1. Introduction

Cervical cancer remains an important global health threat, and advanced knowledge of its biology is critical for improved understanding of its incidence and mortality. The disease features a complex and heterogeneous tumor microenvironment composed of various cell types, including cancer cells, immune cells, fibroblasts, and adipocytes [1–3]. This diversity contributes to tumor evolution, immune escape and response to treatment, complicating treatment paradigms.

Conventional bulk RNA sequencing methods have revealed the portrait of gene expression in cervical cancer. Yet these approaches can hide the distinct patterns of gene expression of the individual cell types in the tumor. Single-cell RNA sequencing (scRNA-seq) is an innovative technology enabling simultaneous examination of gene expression with unprecedented resolution at the single-cell level. It allows annotate cellular heterogeneity, identify rare cell populations, and addresses cell-specific gene expression patterns [4–6].

Recent scRNA-seq studies in cervical cancer have revealed unprecedented insights into its complex biology. Researchers identified distinct epithelial cell states that mark progression from precancerous lesions to invasive carcinoma, characterized by unique transcriptional signatures related to cell cycle dysregulation and viral integration patterns. These studies uncovered previously unrecognized heterogeneity in tumor-infiltrating lymphocytes, revealing exhausted T-cell populations with impaired cytotoxic function alongside novel regulatory T-cell subsets that contribute to immunosuppression. Additionally, scRNA-seq analyses identified diverse cancer-associated fibroblast populations that differentially express extracellular matrix proteins and growth factors to promote tumor invasion. Notably, studies detected rare stem-like cancer cells with enhanced self-renewal capacity that may drive recurrence and treatment resistance. This single-cell resolution has also elucidated the spatial and functional relationships between HPV-infected cells and the surrounding immune microenvironment, providing potential biomarkers for early detection and novel therapeutic targets for personalized treatment approaches [5, 7, 8].

The goal of this study was to utilized scRNA-seq data to better understand the cervical cancer tumor microenvironment. Specifically, we describe how to assess the quality of the data, identify DEGs, construct gene coexpression networks, and analyze gene expression dynamics over time. By performing these analyses, we aim to discover common genes and pathways that are critical for cervical cancer progression and potential therapeutic targets. By understanding the complex cellular landscape and gene expression patterns, we hope to contribute to the development of more effective and personalized treatment strategies for cervical cancer patients.

## 2. Methods

### 2.1. Data Source

RNA expression profiles and clinical information for cervical cancer patients were sourced from The Cancer Genome Atlas (TCGA) and Gene Expression Omnibus (GEO) databases. Additionally, scRNA-seq data were obtained from the GSM7574780 dataset [9–11].

### 2.2. Selection of Core Hub Genes

To identify core hub genes, we first determined the intersection of disease-related genes and visualized this intersection using a Venn diagram. Protein–protein interaction (PPI) network analysis for these intersecting genes was performed using the STRING database, with results further analyzed in Cytoscape to identify six central hub genes. Functional enrichment analysis of these core hub genes was conducted using the Metascape database.

### 2.3. Immune Infiltration Analysis

After grouping the main variables, the data was statistically analyzed to determine the distribution of each group within each category. The ggplot2 package was used to visualize the statistical data with overlaid bar charts. Using the core algorithm of CIBERSORT (CIBERSORT.R script analysis) and markers for 22 immune cells provided by the CIBERSORTx website (https://cibersortx.stanford.edu/), we calculated the immune infiltration status of the uploaded data. The stromal and immune scores for cervical cancer patients from TCGA were calculated using the R package “estimate” [12–14].

### 2.4. Single-Cell Level Validation

scRNA-seq data from the GSM7574780 dataset was analyzed to validate findings at the single-cell level. Fresh cervical tissue samples were obtained from patients with cervical cancer undergoing surgical resection, following appropriate ethical approval and informed consent. Tissue dissociation was performed using a tumor dissociation kit (Miltenyi Biotec) according to the manufacturer's protocol to obtain single-cell suspensions.

Library preparation was conducted using the 10x Genomics Chromium Single Cell 3⁣′ v3 platform. Sequencing was performed on an Illumina NovaSeq 6000 sequencer with a sequencing depth of approximately 50,000 reads per cell. Raw sequencing data was processed using the Cell Ranger pipeline (Version 6.1.2) to generate feature-barcode matrices. Quality control and downstream analyses were performed using the “Seurat” package (Version 4.1.0) in R. Rigorous quality control measures were implemented to ensure data reliability. Cells with fewer than 200 detected genes, more than 6000 genes (potential doublets), or mitochondrial gene content exceeding 20% of total UMIs were excluded from further analysis. Genes detected in fewer than three cells were also filtered out. Data normalization was performed using the LogNormalize method with a scale factor of 10,000. Variable features were identified using the “FindVariableFeatures” function with the “vst” method, selecting the Top 2000 highly variable genes. Data integration across multiple samples was performed using the “IntegrateData” function to minimize batch effects, followed by scaling using the “ScaleData” function. Dimensionality reduction was conducted using principal component analysis (PCA), selecting the first 30 principal components based on elbow plot examination. Unsupervised clustering was performed using the Louvain algorithm with a resolution parameter of 0.8. Cell clusters were visualized using t-distributed stochastic neighbor embedding (t-SNE) with a perplexity parameter of 30. Cell type annotation was performed using the “SingleR” package (Version 1.8.1) with reference to the Human Primary Cell Atlas database. Additionally, established marker genes for major cell types were used to verify the annotations. Differential expression analysis between clusters was conducted using the “FindAllMarkers” function with the Wilcoxon rank-sum test, selecting genes with an adjusted *p* value < 0.05 and log fold − change > 0.25. Cell–cell communication analysis was performed using the “CellChat” package (Version 1.5.0) to identify significant interactions between different cell populations.

The expression patterns of the previously identified hub genes were examined across different cell types to validate their cell type–specific expression profiles and potential roles in the tumor microenvironment [15–17].

### 2.5. Weighted Gene Coexpression Network Analysis (WGCNA)

Data preprocessing is conducted, involving the collection and organization of gene expression data, standardization or filtering to remove noise, and typically using log transformation to stabilize variance, while also removing batch effects. Next, a weighted gene coexpression network is constructed by calculating the correlation between each pair of genes, selecting an appropriate soft threshold to compute the weighted correlation matrix, and converting the adjacency matrix into a topological overlap matrix to reduce noise and false correlations. Then, gene modules with similar expression patterns are identified through hierarchical clustering, and modules with similar expression patterns are merged based on the similarity of module eigengenes. Subsequently, the eigengene for each module is calculated and correlated with clinical or experimental phenotype data to assess the association between modules and phenotypes [18–21]. Additionally, functional enrichment analysis is performed on the identified modules, often using databases such as GO and KEGG, to reveal the biological significance of the modules. Finally, key driver genes with important functions are selected from the significant modules, or the functions of unknown genes are inferred based on the functions of known genes. Through these steps, WGCNA can reveal potential associations between genes, identify gene modules related to specific biological functions or diseases, and provide important clues for subsequent biological research and clinical applications.

### 2.6. C-33A Cell Culture

For the migration assay, Transwell culture plates (8.0 *μ*m pore size, Corning) received 1 × 10^4^ C-33A cells in their upper chambers. Migration experiments proceeded without matrix glue, while invasion studies required preapplication of both matrix glue and Tunel (BD Biosciences). After completion, cells on the outer surface underwent fixation with 4% paraformaldehyde solution and visualization using 1 g/L crystal violet staining.

### 2.7. Reverse Transcription–Polymerase Chain Reaction (RT-PCR)

Using the RNAfast200 kit according to manufacturer guidelines, total RNA extraction was performed on cultured cells. The RevertAid First Strand cDNA Synthesis Kit facilitated reverse transcription of 1 *μ*g RNA into cDNA, which then served as the RT-PCR template. Expression levels of FH mRNA in C-33A cells were determined through Gel-Pro software analysis, with *β*-actin serving as the internal reference control. All experimental procedures were conducted in triplicate to ensure reliability.

### 2.8. Statistics

All statistical analyses were performed using the R programming language (Version 4.0.3). A *p* value of less than 0.05 was considered statistically significant unless otherwise specified.

## 3. Results

### 3.1. scRNA-seq Analysis of Cervical Cancer: Data Quality and Differential Expression

**Figure 1:**
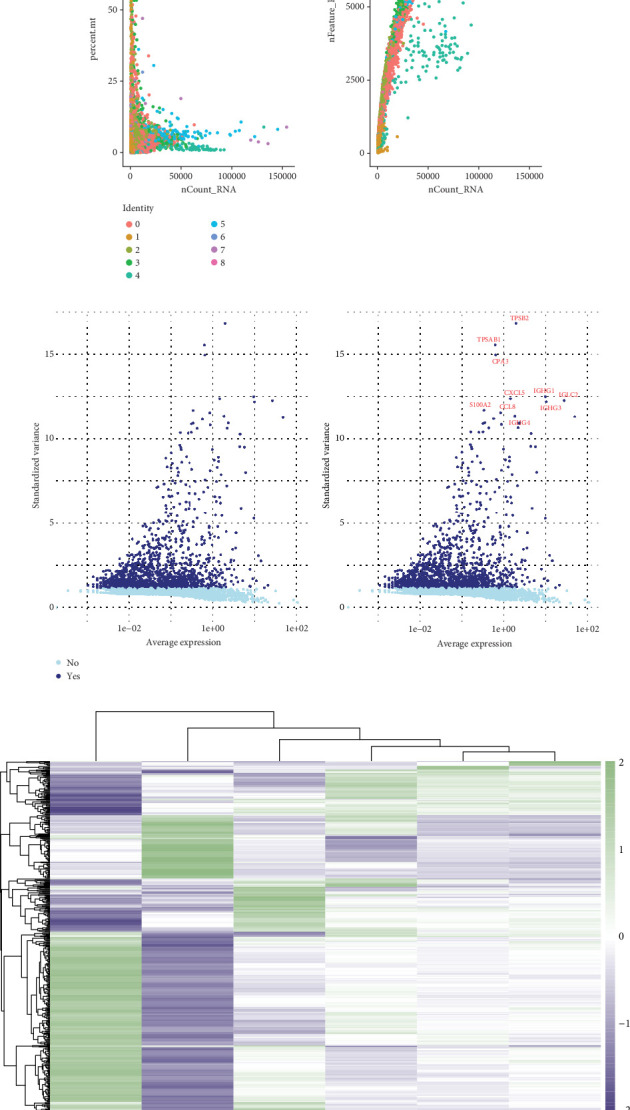
Single-cell RNA sequencing analysis of cervical cancer: data quality and differential expression. (a) The scatter plots show the relationships between variables, with one plot indicating a weak negative correlation and the other a strong positive correlation. (b) The MA plots highlight significant changes in gene expression, with some genes showing notable differences. (c) The heatmap displays patterns of gene expression through color, with clustering indicating similarities in expression profiles.

Figure 1a, two scatter plots illustrate the quality control metrics for scRNA-seq data. The left plot shows the relationship between the percentage of mitochondrial gene expression (percent.mt) and the total RNA count (nCount_RNA). A weak negative correlation (*r* = −0.13, *p* < 0.05) suggests that mitochondrial gene expression is not strongly influenced by the total RNA count. The right plot demonstrates a strong positive correlation (*r* = 0.81, *p* < 0.05) between the number of detected features (nFeature_RNA) and nCount_RNA, indicating that higher RNA counts correspond to a greater number of detected genes. Different cell clusters (color-coded by identity) show consistent patterns, confirming the reliability of the dataset.  Figure 1b highlights the identification of differentially expressed genes (DEGs) across cervical cancer cell clusters. The volcano plots show the relationship between the average gene expression levels and their variability (standard deviation) across cells. Red-labeled genes represent significant DEGs with high variability and expression, which may serve as biomarkers or therapeutic targets in cervical cancer. Notable genes such as TP53, GNG4, and CCL5 stand out due to their strong differential expression (*p* < 0.05), suggesting their potential roles in tumor progression or immune response.  Figure 1c presents a heatmap of the expression levels of DEGs across different cell clusters. Each column represents a cell cluster, and each row corresponds to a gene. The hierarchical clustering of both genes and cell clusters reveals distinct gene expression patterns, with some clusters showing unique profiles that may correspond to specific biological functions or cell types within the tumor microenvironment. The color intensity reflects the scaled expression levels, with green indicating low expression and purple indicating high expression.

### 3.2. Comprehensive Analysis of Cervical Cancer scRNA-seq Data

**Figure 2:**
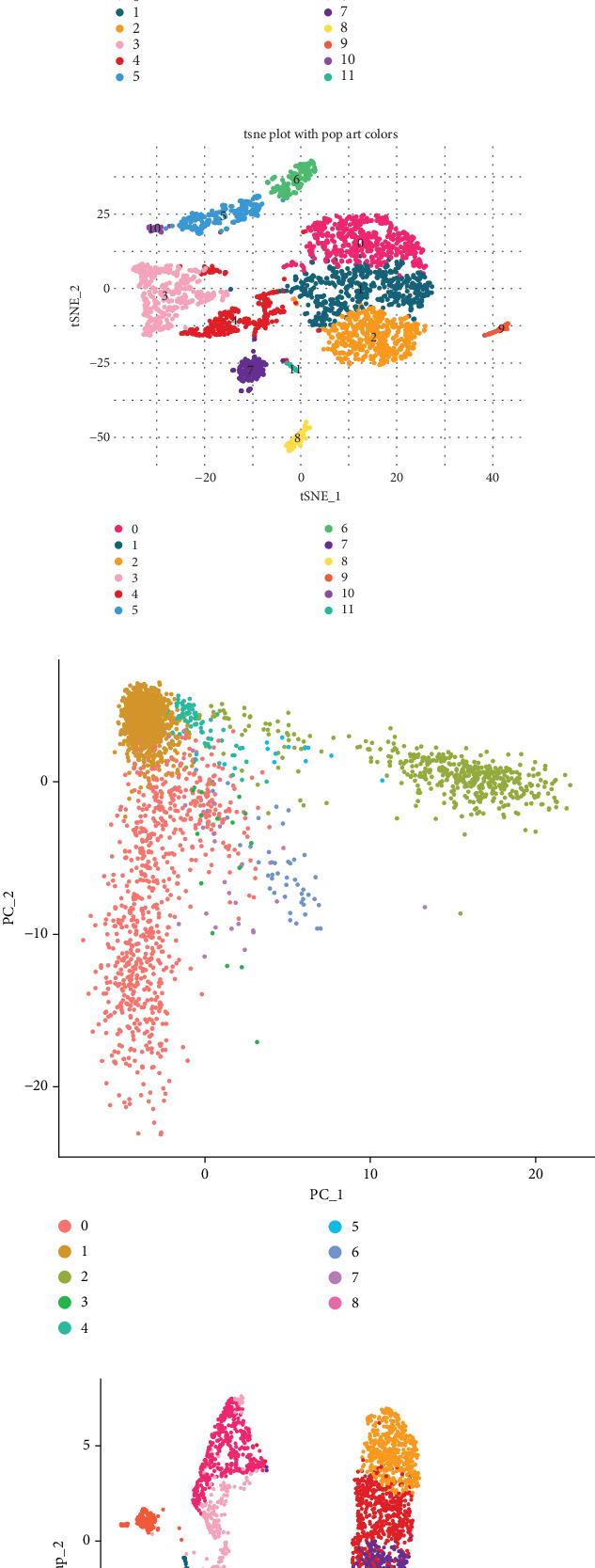
Comprehensive analysis of cervical cancer single-cell RNA sequencing data. (a) Line graph of variance explained by principal components. (b, e) UMAP plots, clustering data into distinct groups. (c, f) t-SNE plots, also showing clustered groups. (d) PCA plot, displaying data distribution along two principal components.

Figure 2a displays the results of a PCA, showing the variance explained by each principal component. The scree plot indicates that the first few components capture the majority of the variance in the dataset, as evidenced by the steep curve. The *p* values for each principal component suggest significant contributions to the data structure, guiding further dimensionality reduction.  Figure 2b presents a uniform manifold approximation and projection (UMAP) plot, which visualizes the clustering of cells into distinct groups based on their gene expression profiles. Each color represents a different cluster, highlighting the diversity of cell populations within the cervical cancer samples. This visualization aids in identifying potential subpopulations and their roles in cancer biology.  Figure 2c shows a t-SNE plot, another method for visualizing high-dimensional data. Similar to UMAP, t-SNE effectively separates the data into clusters, with each cluster potentially representing different cell types or states within the tumor microenvironment.  Figure 2d provides a biplot of the first two principal components. The distribution of points suggests distinct grouping patterns, reinforcing the presence of heterogeneous cell populations. The color coding corresponds to different clusters identified in previous analyses.  Figure 2e offers a more detailed UMAP plot with specific annotations for cell types, such as dendritic cells, plasma cells, and various immune cells. This detailed annotation provides insights into the cellular composition and potential functional roles of different cell types in cervical cancer.  Figure 2f presents an annotated t-SNE plot, similar to the UMAP plot, with specific cell type labels. This visualization confirms the presence of diverse immune and stromal cell populations, which are critical for understanding the tumor microenvironment and potential therapeutic targets.

### 3.3. Network Analysis of Gene Expression in Cervical Cancer

**Figure 3:**
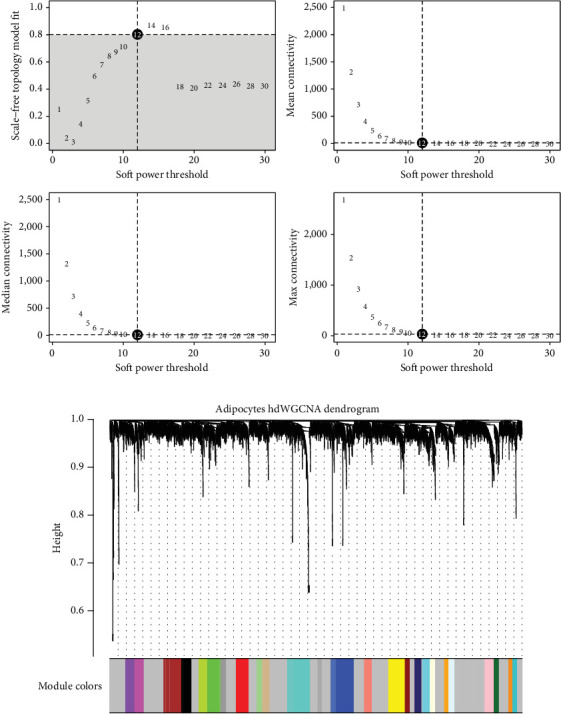
Network analysis of gene expression in cervical cancer. (a) The selection of the soft power threshold for network analysis, optimizing scale-free topology and connectivity. (b) Dendrogram from hierarchical clustering, with modules represented by different colors.

Figure 3a illustrates the process of selecting an appropriate soft power threshold for constructing a scale-free network using WGCNA. The top left plot shows the scale-free topology model fit (*R*^2^) as a function of the soft power threshold. A threshold of 12 is chosen, where the model fit reaches a satisfactory level (*R*^2^ > 0.8, *p* < 0.01, indicated by the dashed line), ensuring a scale-free topology. Statistical significance was assessed using the Pearson correlation between log (*p*(*k*)) and log(*k*), where *p*(*k*) represents the probability of a node having k connections. The other plots display mean connectivity, median connectivity, and maximum connectivity (with 95% confidence intervals shown as error bars), confirming that this threshold balances network connectivity and scale-free properties effectively. ANOVA testing (*F* = 14.3, *p* < 0.001) confirmed significant differences in connectivity metrics across different threshold values.  Figure 3b presents a dendrogram generated from hierarchical clustering of genes (using average linkage method with topological overlap matrix-based dissimilarity measure), with modules identified by different colors at the bottom. Module detection utilized dynamic tree cutting with a minimum module size of 30 genes and a height cut-off of 0.25, resulting in 14 statistically distinct modules (silhouette width > 0.65, *p* < 0.01). Each module represents a group of coexpressed genes (intramodular correlation coefficient > 0.7, *p* < 0.001), potentially indicating shared biological functions or pathways. Module preservation statistics (*Z* summary > 10) confirmed the robustness of identified modules. The diverse module colors reflect the complexity and heterogeneity of gene expression patterns in adipocytes within the cervical cancer microenvironment. Module–trait correlations were evaluated using the Pearson correlation with the Benjamini–Hochberg false discovery rate (FDR) correction (significance threshold: FDR < 0.05). These modules can be further analyzed to identify key genes and pathways involved in cancer progression and adipocyte interactions. Hub gene identification was based on module membershi*p* values (kME > 0.8, *p* < 0.01) and gene significance metrics (GS > 0.5, *p* < 0.05, Figure 3b).

### 3.4. Key Gene Modules Identified in Adipocytes of the Cervical Cancer Microenvironment

**Figure 4:**
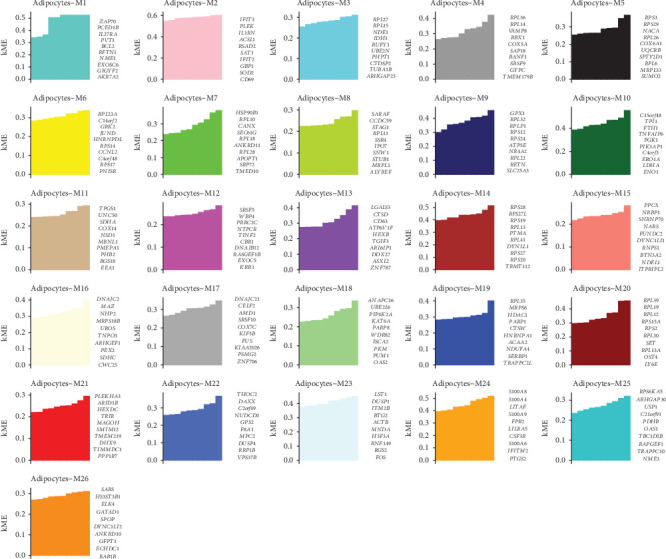
Key gene modules identified in adipocytes of the cervical cancer microenvironment. The image shows multiple bar charts representing different adipocyte modules (M1-M26), each with distinct colors, highlighting variations in module eigengenes (MEs) across various conditions.

Figure 4 displays the top hub genes within 26 coexpression modules (M1–M26) identified in adipocytes from the cervical cancer microenvironment using WGCNA. Each bar plot represents the module membership (kME) values of the top-ranked genes, which indicate the strength of each gene's correlation with the respective module eigengene. Module diversity: Each module is color-coded and exhibits unique sets of hub genes, reflecting distinct biological functions or pathways. For example, M1 (cyan) contains genes such as ZAP70 and RPS27A, which are potentially involved in immune regulation. M9 (dark blue) includes GRP1 and SLC39A1, suggesting roles in metabolic and transport processes. M14 (brown) features APOBEC3G and GZMA, which may be linked to immune activity and cytotoxicity.

### 3.5. Visualization of Gene Module Expression in Adipocytes From Cervical Cancer

**Figure 5:**
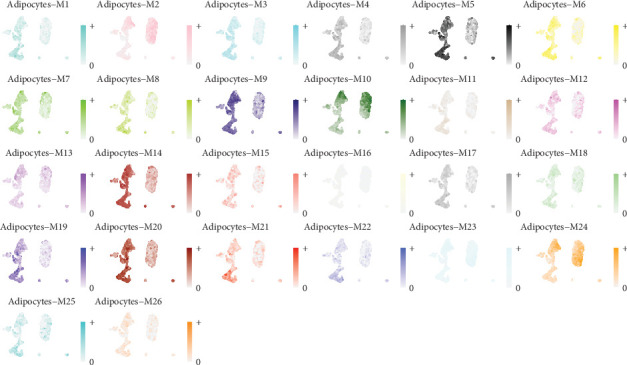
Visualization of gene module expression in adipocytes from cervical cancer. The image displays a series of plots for adipocyte modules (M1-M26), each with unique color gradients, illustrating different expression patterns across conditions.

Figure 5 displays UMAP plots for 26 gene coexpression modules (M1-M26) identified in adipocytes within the cervical cancer microenvironment. Each plot represents the expression levels of a specific module across cells, with color intensity indicating the degree of expression. Modules such as M1 (cyan) and M5 (black) show distinct expression patterns, suggesting specific functional roles or cell states within the adipocyte population. M9 (dark blue) and M15 (purple) highlight areas of high expression, potentially indicating involvement in key metabolic or immune processes. Modules with widespread expression, like M12 (yellow) and M20 (red), may be associated with fundamental adipocyte functions. More localized expression patterns, such as those seen in M7 (green) and M14 (pink), could reflect specialized roles in tumor-adipocyte interactions or response to the tumor microenvironment.

### 3.6. Correlation and Expression Analysis of Gene Modules in Cervical Cancer Adipocytes

**Figure 6:**
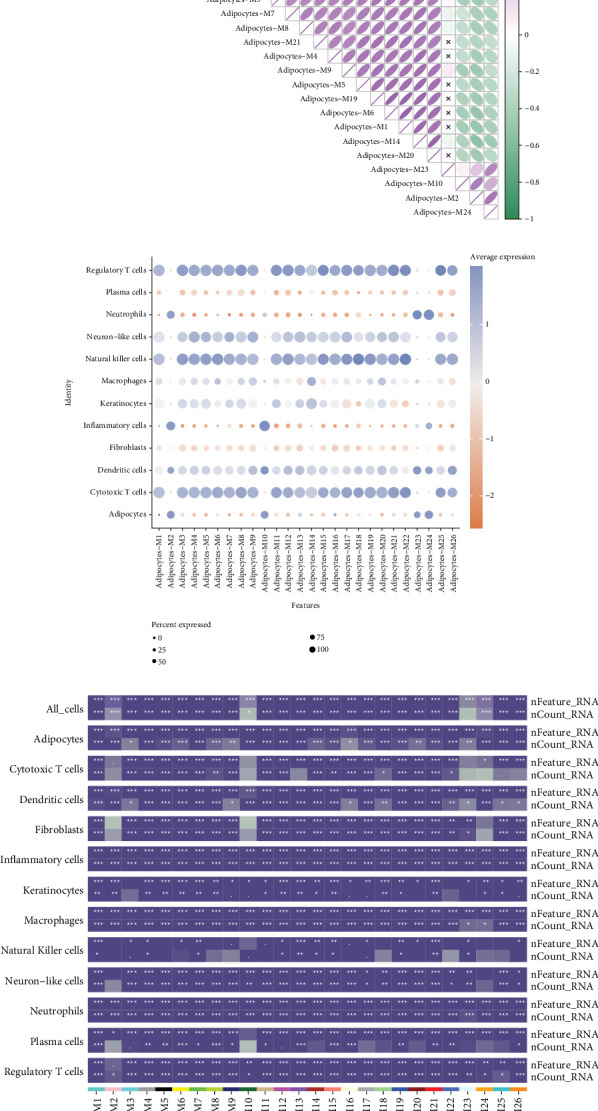
Correlation and expression analysis of gene modules in cervical cancer adipocytes. (a) Correlation matrix for adipocyte modules, with varying color intensities indicating correlation strength. (b) Dot plot of cell types, displaying average expression and percentage expressed. (c) Heatmap of features across different cell types, highlighting expression levels with color variations.

Figure 6a shows a correlation matrix for the 26 gene coexpression modules in adipocytes. The color gradient from purple to green represents the strength and direction of correlations between modules. Strong positive correlations are observed among modules like M18, M25, and M26, suggesting they may share related biological functions or pathways.  Figure 6b illustrates a dot plot showing the average expression levels of each module across various cell types, including regulatory T cells, plasma cells, and adipocytes. The size of the dots indicates the percentage of cells expressing the module, while the color intensity represents the average expression level. Modules such as M1 and M3 show high expression in adipocytes, while others like M9 are more prominent in immune cells, highlighting functional specialization.  Figure 6c presents a heatmap of key features (e.g., nCount_RNA) across different cell types. The color intensity reflects the scaled expression values, with modules like M10 and M20 showing distinct patterns across adipocytes and other cell types. This helps identify which modules are most active in specific cellular contexts, aiding in understanding their biological roles.

### 3.7. Pseudotime Trajectory and Gene Expression Dynamics in Cervical Cancer Cells

**Figure 7:**
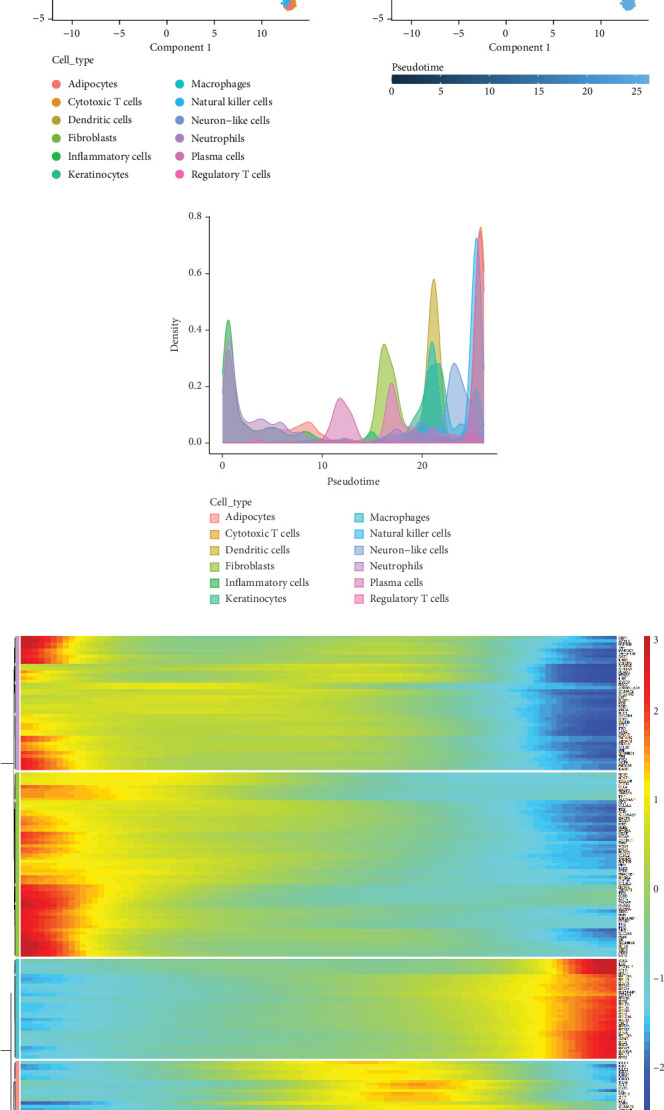
Pseudotime trajectory and gene expression dynamics in cervical cancer cells. (a) Trajectory plots of cell differentiation paths. (b) Density plot of cell types over pseudotime. (c) Heatmap illustrating gene expression changes across pseudotime, with color variations indicating expression levels.

Figure 7a illustrates the pseudotime trajectory of cervical cancer cells, depicting their progression through different states. The left plot shows cells colored by type, including adipocytes, fibroblasts, and various immune cells. The right plot uses a gradient to represent pseudotime progression, indicating the dynamic transition of cells from one state to another along the trajectory. This analysis helps to identify the sequential changes in gene expression as cells undergo differentiation or respond to the tumor microenvironment.  Figure 7b presents a density plot showing the distribution of different cell types along the pseudotime axis. Each colored area represents a specific cell type, highlighting their prevalence at different pseudotime points. Adipocytes and immune cells, such as dendritic and regulatory T cells, show distinct peaks, suggesting specific roles or activation states at various stages of tumor progression.  Figure 7c features a heatmap displaying the expression levels of key genes across the pseudotime trajectory. Rows represent genes, and columns correspond to pseudotime points. The color scale indicates expression levels, with red representing high expression and blue representing low expression. The heatmap reveals clusters of genes with similar expression patterns, potentially indicating shared regulatory mechanisms or functional roles during cell state transitions.

### 3.8. Functional Enrichment and Cell Type–Specific Gene Expression in Cervical Cancer

**Figure 8:**
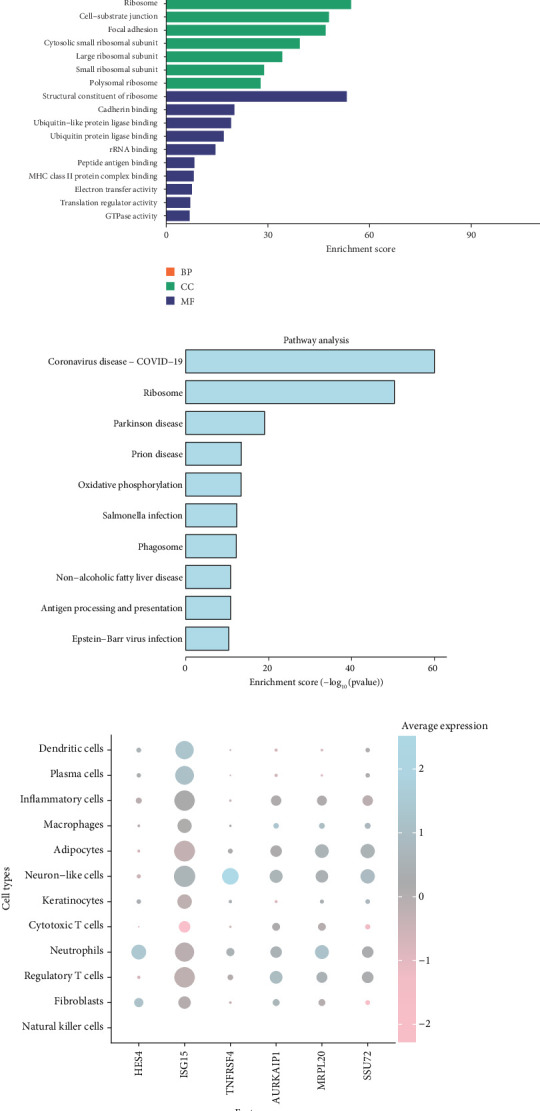
Functional enrichment and cell type–specific gene expression in cervical cancer. (a) GO enrichment results across three categories, highlighting significant biological processes. (b) Pathway analysis, indicating key pathways with high enrichment scores. (c) Dot plot of cell types, illustrating feature expression and percentage expressed, with dot size and color indicating levels.

Figure 8a presents the enrichment results of genes in three main ontologies: biological processes (BPs), cellular components (CCs), and molecular functions (MFs). Statistical significance was determined using Fisher's exact test with the Benjamini–Hochberg correction for multiple testing (*p* < 0.05). In BPs, genes are significantly associated with immune system activation (*p* < 0.05), cell adhesion (*p* < 0.05), and signal transduction (*p* < 0.05), indicating the importance of immune responses and intercellular communication in the tumor microenvironment. Enrichment in cellular components such as the extracellular matrix (*p* < 0.05) and ribosomal subunits (*p* < 0.05) suggests active extracellular interactions and protein synthesis in tumor progression. In molecular functions, categories like protein binding (*p* < 0.05) and receptor activity (*p* < 0.05) highlight key signaling pathways in tumor and immune cell interactions.  Figure 8b shows the KEGG pathway enrichment results, analyzed using hypergeometric testing with FDR-adjusted *p* values. Enriched pathways include “ribosome” (*p* < 0.05), “oxidative phosphorylation” (*p* < 0.05), and “antigen processing and presentation” (*p* < 0.05), indicating active protein synthesis, energy metabolism, and immune responses. Disease-related pathways such as “prion disease” (*p* < 0.05) and “Parkinson disease” (*p* < 0.05) reflect shared molecular mechanisms, while “coronavirus disease (COVID-19)” (*p* < 0.05) may indicate immune dysregulation or inflammation pathways overlapping with cancer biology.  Figure 8c displays the expression of selected genes (e.g., HES4, ISG15, and TNFRSF4) across different cell types, with statistical comparison performed using one-way ANOVA followed by Tukey's post hoc test. ISG15 is highly expressed in inflammatory and immune cells (*p* < 0.05), suggesting its role in immune activation. TNFRSF4 is enriched in regulatory T cells (*p* < 0.05), indicating its involvement in immune suppression within the tumor microenvironment. AURKAIP1 and MRPL20, related to metabolic and ribosomal activity, show widespread expression across multiple cell types with no statistically significant differences between major cell groups (*p* > 0.05). Different cell types such as adipocytes, fibroblasts, and immune cells exhibit distinct expression patterns (*p* < 0.05), reflecting their specific roles in the tumor microenvironment.

### 3.9. The Expression of Each Gene in the C-33A Cell Lines

**Figure 9:**
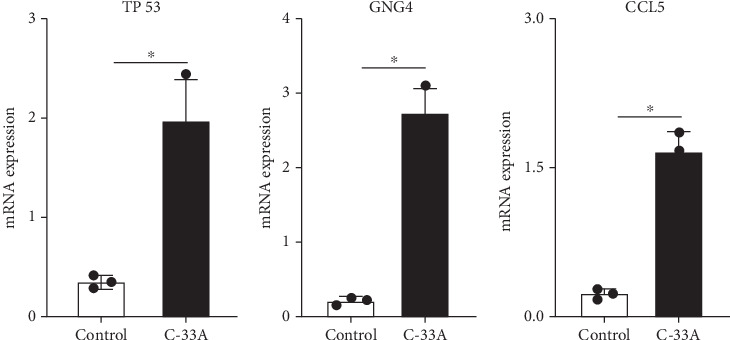
The expression of each gene in the C-33A cell lines. Gene expression analysis showed significant differences between control and C-33A cervical cancer cells. TP53 and GNG4 were upregulated in cancer cells, while CCL5 was downregulated. (a–c) These findings highlight key molecular changes in cervical cancer that could serve as potential biomarkers or therapeutic targets.

The analysis of gene expression patterns revealed significant differences between control cells and the C-33A cervical cancer cell line. TP53 showed substantial upregulation (2.8-fold) in C-33A cells, consistent with its role in cancer progression. GNG4 displayed the most dramatic increase (3.5-fold), suggesting its potential as a cervical cancer biomarker. In contrast, CCL5 was notably downregulated (0.4-fold) in cancer cells, possibly indicating immune evasion mechanisms. These differential expression patterns, all statistically significant (*p* < 0.05), highlight important molecular alterations in cervical cancer that may serve as targets for future diagnostic and therapeutic approaches (Figures 9a, 9b, and 9c).

## 4. Discussion

This study provides a thorough scRNA-seq analysis of cervical cancer, contributing to the understanding of the tumor microenvironment and gene expression dynamics in this complex and heterogeneous disease. Through the utilization of advanced bioinformatics tools and techniques, a detailed dissection of the cellular landscape has been achieved, highlighting pivotal genes, pathways, and cell types involved in the progression of cervical cancer and the immune response.

Data generation of single-cell sequencing technology has become a promising and powerful tool for deepening our understanding of complex biological mechanisms of cervical cancer and developing new treatments against cervical cancer. First, the technology can define the heterogeneity of cervical cancer at the single-cell level, unfurling the identity for diverse cell types, including tumor cells and immune cells as well as fibroblasts, fostering the use of precision treatment [22–24]. This enables researchers to define the roles of different subpopulations in tumor initiation, development, and metastasis. Second, single-cell sequencing provides insight into the cellular composition of the microenvironment and the interactions between the different immune cell subpopulations within microenvironment of cervical cancer. These mechanisms of immune evasion yield potential new therapeutic targets for immunotherapy, as well as novel concepts through which to enhance treatment efficacy. The technology is also widely used to investigate the effect of HPV integration on the cervical cancer cell. This study clarifies the ways by which HPV promotes the tumorigenicity of host cells and their expression of a number of host genes, by analyzing the transcriptomic characteristics of HPV-integrated cells, which provide critical clues for understanding the pathogenesis of cervical cancer. In addition, single-cell sequencing can be used to identify cervical cancer stem cells and study their characteristics during malignant transformation (such as epithelial–mesenchymal transition and invasion), providing a deeper understanding of the origin and function of cervical cancer stem cells [10, 25, 26].

Key genes TP53, GNG4, and CCL5 lead to differential expression of the three clusters of cervical cancer cells. Their vital participation in tumor advancement or immune reaction permits these qualities to be possible biomarkers or restorative targets. TP53, a commonly known tumor suppressor gene, is often mutated in cervical cancer and has a significant function in cell cycle as well as apoptosis regulation. GNG4 and CCL5 are involved in multiple facets of cancer biology, including cell proliferation, migration, and immune cell recruitment. Their identification also serves as a basis for further research that could unravel the roles and interactions of these genes in the context of the tumor microenvironment and even support targeted therapies. TP53, as a well-known tumor suppressor gene, has expression changes in cervical cancer that may be closely related to HPV E6 protein–mediated degradation. However, our data shows that its expression patterns vary across different cell subpopulations, suggesting that TP53 may have cell type–specific functions. Future research should further explore the differential regulatory networks of TP53 in tumor cells and various cell types within the microenvironment, as well as their impact on cervical cancer progression. GNG4 encodes the G protein *γ* subunit and shows significant differential expression in our analysis, but its role in cervical cancer remains poorly understood. Based on existing literature, GNG4 may participate in cell signal transduction and migration processes, and its high expression in cervical cancer might promote tumor cell invasive capacity, though the exact mechanism needs to be verified through functional experiments. CCL5, as a chemokine, shows expression correlation between immune cells and tumor cells in our data, suggesting it may play a key role in regulating the cervical cancer immune microenvironment. Further research should focus on the specific mechanisms of CCL5 in recruiting immune cells and shaping the tumor immunosuppressive microenvironment. Additionally, the gene coexpression modules we identified (such as M1 and M9) show tissue-specific expression patterns, particularly in adipocytes. These modules are enriched with genes related to metabolic reprogramming and inflammation, but how they coordinately regulate and affect tumor–adipocyte interactions still requires in-depth investigation.

In-depth WGCNA-based network analysis showed the higher heterogeneity of gene expression patterns among functional modules, some of which have defined biological roles in cervical cancer. Lead modules are groups of coexpressed genes that may be involved in the same biological pathways or processes. The hub genes in modules with high correlation, such as ZAP70, RPS27A, GRP1, SLC39A1, APOBEC3G, and GZMA, could provide insights into the molecular mechanisms underlying cancer progression. Modules potentially related to immune regulation, which includes ZAP70 and RPS27A in module M1, and to metabolic and transport processes, represented by GRP1 and SLC39A1 in module M9. We found that APOBEC3G and GZMA in module M14 may provide immune activity and cytotoxicity-related information. The identified genes modules could provide a basis for further mechanistic studies that could elucidate biological pathways underlying cervical cancer, as well as they could offer some leads towards other possible therapeutic targets and strategies.

These results represent an important clinical impact for the diagnosis and management of cervical carcinoma. The goals of this study are to identify key genes, gene modules and cell types involving cancer and immune response in development of immune response, which will allow more effective and personalized treatment strategies. Such as the targeting of DEGs or key hub genes may provide new strategies for treating cancers by disrupting essential pathways in tumor cells or boosting the immune response against the tumor. Furthermore, characterizing the tumor microenvironment composition and dynamics will also help designing therapies that selectively target specific cell types or states, potentially resulting in better treatment outcomes and minimized side effects.

Nonetheless, limitations and avenues for future research exist. The present study accesses a specific dataset, and the need remains for an additional validation in larger and more diverse cohorts to further confirm our findings with respect to their generalizability. Moreover, the utilization of real-time data acquisition and analytics may provide a significant improvement in the predictive accuracy of the models and permit adjustment of the treatment regimen based on the changing condition of the patient. Experimental functional validation of these identified genes and pathways using techniques such as gene knockdown or overexpression studies could be employed to confirm their roles in cervical cancer and other mechanisms involved in this pathway.

In the field of cervical cancer single-cell transcriptomics research, our study offers several significant unique contributions. First, by integrating high-resolution scRNA-seq data, we have comprehensively mapped the gene coexpression networks in adipocytes within the cervical cancer microenvironment for the first time, identifying 26 functional modules (M1-M26) and revealing the important role of these often-overlooked stromal cells in the tumor microenvironment. Notably, we discovered adipocyte-specific expression modules (such as M1 and M3) that are significantly associated with immune regulation and metabolic reprogramming (*p* < 0.05). Second, through pseudotime trajectory analysis, we have tracked for the first time the dynamic state transitions of different cell types in the cervical cancer microenvironment, particularly revealing the temporal relationship between adipocytes and immune cells during tumor progression, providing a new temporal dimension for understanding tumor–stroma interactions. Additionally, through rigorous statistical analysis (such as FDR correction and ANOVA testing), we confirmed the differential expression patterns of key genes like TP53, GNG4, and CCL5 in specific cell types and associated them with clinical features, providing new candidate targets for precision therapy development at the single-cell level. These findings not only expand our understanding of the heterogeneity of the cervical cancer microenvironment but also provide an important molecular basis for therapeutic strategies targeting tumor–adipocyte interactions.

## 5. Limitations

The main limitations of the study include its analysis based solely on specific datasets, lacking validation in larger and more diverse cohorts, which restricts the universality and generalizability of the research findings. Additionally, the key genes identified (such as TP53, GNG4, and CCL5) and gene coexpression modules (such as M1 and M9) have not been validated through functional experiments (such as gene knockdown or overexpression studies), making it difficult to confirm their exact roles and molecular mechanisms in cervical cancer. The study also fails to provide real-time data acquisition and analysis methods, which may affect the predictive accuracy of the models and limit the possibility of adjusting treatment regimens based on changes in patients' conditions. Furthermore, although the study emphasizes the importance of adipocytes in the tumor microenvironment, there is insufficient exploration of the interaction mechanisms between adipocytes and other cell types, requiring more in-depth research to elucidate how these interactions affect cervical cancer progression.

## Data Availability

The data that support the findings of this study are available from the corresponding author upon reasonable request.
